# Cellular and Molecular Mediators of Bone Metastatic Lesions

**DOI:** 10.3390/ijms19061709

**Published:** 2018-06-08

**Authors:** Giulia Battafarano, Michela Rossi, Francesco Marampon, Andrea Del Fattore

**Affiliations:** 1 Bone Physiopathology Group, Multifactorial Disease and Complex Phenotype Research Area, Bambino Gesù Children’s Hospital, 00146 Rome, Italy; giulia.battafarano@opbg.net (G.B.); michela1.rossi@opbg.net (M.R.); 2 Department of Radiotherapy, Policlinico Umberto I, Sapienza University of Rome, 00146 Rome, Italy; f.marampon@gmail.com

**Keywords:** bone metastasis, osteoclast, osteoblast

## Abstract

Bone is the preferential site of metastasis for breast and prostate tumor. Cancer cells establish a tight relationship with the host tissue, secreting factors that stimulate or inhibit bone cells, receiving signals generated from the bone remodeling activity, and displaying some features of bone cells. This interplay between tumor and bone cells alters the physiological bone remodeling, leading to the generation of a vicious cycle that promotes bone metastasis growth. To prevent the skeletal-related events (SRE) associated with bone metastasis, approaches to inhibit osteoclast bone resorption are reported. The bisphosphonates and Denosumab are currently used in the treatment of patients affected by bone lesions. They act to prevent or counteract the SRE, including pathologic fractures, spinal cord compression, and pain associated with bone metastasis. However, their primary effects on tumor cells still remain controversial. In this review, a description of the mechanisms leading to the onset of bone metastasis and clinical approaches to treat them are described.

## 1. Introduction

Bone is the third most frequent site of metastasis after lung and liver and typically indicates a short-term prognosis in cancer patients [1,2]. Prostate and breast cancers are responsible for up to 70% of skeletal metastasis [3].

The overall incidence of bone metastasis is unknown, but the relative incidence of bone metastasis by tumor type is 65–75% in breast cancer, 65–75% in prostate cancer, 60% in thyroid cancer, 40% in bladder cancer, 20–25% in renal cell carcinoma, and 14–45% in melanoma [1,4]. Patients’ survival after a diagnosis of bone metastasis also depends on the primary tumor: 48 months for thyroid tumors, 19–25 for breast cancer, 12–53 for prostate cancer, 12 for renal cell carcinoma, 6–9 for bladder cancer, 6–7 for lung cancer, and 6 for melanoma [4].

Bone metastasis causes morbidity with severe pain, impaired mobility, fractures, spinal cord compression, bone marrow aplasia, and hypercalcemia [4]. The bone metastasis-associated pain is a progressive condition. Most patients initially experience intermittent dull aches, then the pain becomes progressively more constant and severe. Its degree cannot be predicted by the tumor type, size, and number of metastasis and it is not proportionate to bone involvement [5]. The origin of bone pain could be related to either inflammatory or mechanical components. Indeed, the inflammatory one is due to cytokines and molecules locally released by the tumor and inflammatory cells, including prostaglandins, endothelins, and nerve growth factors; these factors are detected by receptors of the bone sensory neurons leading to pain response or pain sensitization. The mechanical origin of pain is associated to the pressure of the tumor mass within the bone [6].

Pathologic fractures occur in 10–30% of all cancer cases with bone metastasis. Breast cancer accounts for 60% of the pathologic fractures, while lung cancer only for 10% of cases [3]. The most frequent site is the proximal part of long bones, with femur involvement in more than 50% of patients [4]. Rib fractures and vertebral collapses are also very common and can lead to kyphoscoliosis and restrictive lung disease. The extent of the metastatic involvement increases the probability to develop pathological fractures [3]. The deterioration of the mechanical properties of tumor-affected bones is a prerequisite for fracture occurrence. If, in osteolytic lesion, there is bone loss that obviously leads to the impairment of bone mechanical properties, the mechanisms underlying this defect in osteosclerotic lesions is poorly characterized. It was recently demonstrated that in PCa (Prostate cancer)-associated metastasis, the reduction of the alignment degree of collagen fibrils and biological apatite can impair bone mechanical properties [7].

Hypercalcemia is the most common metabolic complication of bone metastatic disease and frequently occurs in lung squamous cell carcinoma, breast cancer, multiple myeloma, and lymphoma [8]. Hypercalcemia is classified in: mild hypercalcemia, when serum calcium level is 10.5–11.9 mg/dL, moderate hypercalcemia, if the calcemic concentration is 12–13.9 mg/dL, and severe hypercalcemia, with blood calcium concentration higher than 14 mg/dL. Mild and moderate hypercalcemia can be asymptomatic or associated with lethargy and musculoskeletal pain, whereas severe hypercalcemia rapidly progresses, leading to gastro-intestinal, renal, and central nervous system dysfunctions, the latter ranging from altered mental status to coma. Finally, hypercalcemia leads to cardiac arrythmias and renal failure [1]. The prognosis in the presence of hypercalcemia is very poor, with a median survival of 10–12 days [4]. The reasons by which hypercalcemia is associated with malignancy are increased parathyroid hormone-related peptide (PTHrP), ectopic or primary PTH secretion, excess of extrarenal activated vitamin D, and multiple concurrent etiologies. Indeed, hypercalcemia is due to focal and generalized osteolysis induced by the tumor cells, increased tubular calcium reabsorption by the kidney, and impaired renal function [9]. 

When one of these signs and symptoms occurs, a basic screen needs to be performed. Complete blood cells count, levels of serum calcium and phosphorous, 25-hydroxyvitamin D, alkaline phosphatase (ALP), creatinine, thyroid-stimulating hormone (TSH), PTH have to be determined, and protein electrophoresis has to be performed to analyze bone turnover and identify hypercalcemia. The analysis of all these parameters needs to be combined with imaging data, including bone scintigraphy, radiographs, magnetic resonance imaging (MRI), and positron emission tomography (PET) [9].

## 2. Metastasis Cascade

The metastasis cascade is a multistep mechanism that leads a primary tumor cell to leave its tissue of origin, come into the circulation, losing its epithelial characteristics and becoming a disseminated tumor cell, survive in the blood, escaping from innate immune, and choose its final destination. Once in a secondary tissue, disseminated tumor cells survive in a dormancy state until a trigger switches them into a proliferating state, inducing metastasis colonization [10]. Valastyan and Weinberg [11] accurately described the complex metastasis cascade steps as follow:

1. Local invasion indicates a primary tumor cell’s ability to entry the surrounding stroma and, then, the neighboring healthy tissue [11]. Initially, carcinoma cells breach the basement membrane, impairing physical structured compartments and liberating tethering growth factors contained in the extracellular matrix, by proteolytic activity. There are two types of invasion: collective invasion, whereby cohesive multicellular units can locally invade, and single-cell invasion characterized by carcinoma cells undergoing mesenchymal or amoeboid invasion programs [12]. In response to microenvironmental signals, tumor cells can switch between these two programs. For single-cell invasion, epithelial–mesenchymal transition (EMT) is required to dissociate a tumor cell from its neighbors. EMT involves the dissolution of E-cadherin-containing junctions and it is orchestrated by several transcription factors, such as Slug, Snail, Twist, ZEB1, and ZEB2 [13]. The loss of the basement membrane allows carcinoma cells to raid the stromal compartment [14].
  After the invasion and progression of a tumor, the stroma becomes “reactive” and inflamed. The inflamed microenvironment enhances the aggressive behavior of tumor cells, establishing a self-amplifying positive feedback loop: carcinoma cells stimulate an inflammatory environment, and inflammation enhances the malignant traits of primary tumor cells. Once in the stromal compartment, carcinoma cells have more opportunities to access the circulation and, thus, to disseminate [15].
2. Intravasation is the process by which locally invasive carcinoma cells enter the lymphatic or, mainly, blood vessels [11]. It should be relevant for clinical practice to clarify how frequently cancer cells spread to the bone marrow through the lymphatic vessels. Indeed, lymph node involvement is useful to enlighten and guide the course of a treatment in breast cancer patients [16]. Moreover, the structural features of tumor-associated blood vessels strongly influence intravasation [17].
3. Once in the lumen of blood vessels, carcinoma cells disseminate through the circulation, and circulating tumor cells represent a metastatic intermediate. They need to face several stresses: initially, matrix detachment, that induces anoikis in epithelial cells [18]; secondly, haemodynamic stress and innate immune system predation [19]. Carcinoma cells bypass both of the latter stresses through large emboli formation via the interaction with blood platelets. Once coated by platelets, tumor cells are more able to persist in the circulation [11].
4. Arrest at a distant organ site and extravasation. Actually, it is debated if circulating tumor cell arrest is simply guided by a passive process, whereby tumor cells are trapped in the capillary bed, or by homing ligand–receptor interactions with the microvasculature in specific organs. In the microvasculature of a target organ, tumor cells grow in the lumen or can cross the endothelial and pericytes layer by the extravasation process, invading the parenchyma. For long time, it has been postulated that extravasation is the reverse process of intravasation. Instead, extravasation may be quite different mechanistically. For example, tumor-associated macrophages (TAMs) that help intravasation are not equally available to facilitate extravasation [20,21]. In addition, the neovasculature formed around the primary tumor and used by tumor cells to disseminate in the circulation is very different from the normal vasculature in a distant tissue [17]. It seems to be plausible that the specific microenvironment in a distant tissue may play a role, supporting extravasation.
  The preferential choice of a primary tumor to metastasize to specific organs is described in two theories, namely, the Paget’s and the Edwin’s theories [22,23,24]. The first one is also known as the “seed and soil theory” and indicates the ability of circulating tumor cells to colonize specific organs. Indeed, Paget proposed that the cancer cells, being the “seeds”, only metastasize to that site, the “soil”, where they can adapt for survival and proliferation [23,24]. The Edwin’s theory is based on the concept that tissue tropism is simply associated with anatomic reasons and reflects a passive physical trapping of the circulating tumor cells within the capillary beds of the first organ they go through [22]. None of these two theories seems to be sufficient to explain the preferential choice of a specific tissue where to metastasize. Indeed, the weak point of Paget’s theory is that, actually, it is unknown why a tumor derived from one of two paired organs does not colonize the contralateral one. On the other hand, the Edwin’s theory cannot explain why breast cancer cells rarely colonize the heart, through which they pass [25].
  Bone is a fertile soil for metastasis. Indeed, it supplies a unique environment for metastasis represented by the hematopoietic marrow in long bones and axial skeleton for which breast cancer cells have a predilection [26]. Moreover, the bone marrow may provide the premetastatic niche [27]. Anatomic features, such as abundant sinusoids and slow blood flow, make the bone favorable for metastasis [26]. Finally, bone turnover provides available resources for tumor cells [28]. The bone matrix is a storage of growth factors that, during remodeling, are released and may promote homing, colonization, and initial proliferation of tumor cells. This is the basis for the “vicious cycle” of tumor growth. Specifically, tumor cells settle down in bone tissue, where bone remodeling continuously provides them with abundant resources; in turn, the establishment of tumor cells in bone tissue affects bone remodeling, leading to uncoupled resorption and formation and, thus, to the switch from a virtuous cycle to a vicious cycle. The vicious cycle promotes tumor cells growth and bone lesion onset [29].
5. Once reached the metastatic locus, tumor cells need to survive in the new microenvironment in order to form micrometastases. This new microenvironment is very different from the primary tumor site and, at least initially, cancer cells are poorly adapted to it. Thus, tumor cells deploy complex mechanisms to survive: on one hand, they modify the foreign microenvironment via the establishment of a “premetastatic niche” [30], which consists in predisposing changes of the distant microenvironment into a more hospitable site for their future settling of; on the other hand, disseminated tumor cells use cell-autonomous programs in order to adapt to the new home [11].

## 3. Types of Bone Metastases

Bone metastases are classified in three types: osteolytic, sclerotic, and mixed metastasis according to the radiographic and/or pathologic appearance of the lesions [31]. This classification depends on the alteration of the bone remodeling activity induced by the tumor cells.

The skeleton is a dynamic and metabolically active organ that undergoes continuous remodeling throughout life [32]. The ability of bone to sustain the loads applied on it, to adjust its architecture to adapt to mechanical stress, and to provide calcium if needed, are conferred from its capability to constantly remodel itself [32,33]. The remodeling involves tightly coupled osteoclasts’ and osteoblasts’ activities. Bone remodeling is a highly ordered process that starts by an essential first step which is the selection of the site to be remodeled. Indeed, the remodeling takes place only where old or damaged bone needs to be replaced. Once the site is established, the osteocytes orchestrate bone remodeling, via mechanisms until now unclarified [34]. Osteoclast precursors are recruited from the marrow on the bone surface, fuse, becoming giant multinucleated cells, and mature in polarized osteoclasts with a basal membrane compartment known as ruffled border appointed to the resorption [35]. During bone resorption, the osteoclasts release from the bone matrix several factors, including Transforming Growth Factor beta (TGF-β), Fibroblast Growth Factor (FGF), Insulin Growth Factor (IGF), Platelet-Derived Growth Factor (PDGF), and Bone Morphogenetic Proteins (BMP) [36]. These molecules are able to stimulate the differentiation of mesenchymal stem and stromal cells (MSC) into osteoblasts and the deposition of bone matrix. Moreover, the osteoclasts are able to stimulate osteoblast differentiation and activity by a bone resorption-independent mechanisms, through the release of secreted factor called “clastokines”, including Hepatocyte Growth Factor (HGF), PDGF, Sphingosine-1 Phosphate) (S1P), and TRAcP [37]. Indeed, osteoblasts mesenchymal precursors are recruited to the previous resorbed bone surface and begin the osteoblastogenesis [36]. Osteoblasts come from the MSC in the marrow and from pericytes and differentiate into fully bone-forming osteoblasts [34]. Mature cuboidal osteoblasts set down osteoid that will be mineralized. Moreover, osteoblasts secrete cytokines essential for the differentiation, survival, and activity of osteoclasts, such as Macrophage Colony-Stimulating Factor (M-CSF) and Receptor Activator of Nuclear factor κ-Β Ligand (RANK-L) [36]. Following the binding of M-CSF to the receptor c-fms (Colony-stimulating factor-1 receptor) on the cell surface of osteoclast precursors, the resulting signaling induces their proliferation and the expression of the receptor RANK, leading to osteoclast differentiation [38]. The interaction of RANK-L with its receptor stimulates the fusion of osteoclast precursors to generate mature multinucleated cells and promotes the survival and activity of osteoclasts [38,39]. Osteoblasts and stromal cells release also Osteoprotegerin (OPG), a decoy receptor for RANK-L that competes for RANK binding and inhibits osteoclastogenesis [38].

During bone remodeling, the perfect combination of bone cells ensures that the resorbed bone is completely replaced by new bone [33]. When the mechanism of bone remodeling is uncoupled, there is a pathologic condition. The coupling is maintained by a number of factors, such as circulating hormones (PTH and 1,25-dihydroxyvitamin D3) and locally generated cytokines, but also matrix-derived signals, secreted by multiple cellular sources, even from the bone marrow [34].

### 3.1. Osteolytic Lesions

Osteolysis is the most common manifestation of bone metastasis. Evidence suggests that osteolytic metastasis pathogenesis and progression are based on the existence of a “vicious cycle” derived from the perturbance of the physiologic “virtuous cycle” characterizing bone turnover [31]. This complex vicious cycle involves mutual interactions among tumor cells, bone cells (osteoclasts and osteoblasts), and the bone matrix. Once established in bone, the tumor cells secrete soluble factors promoting osteoclast differentiation and resorption. Growth factors are mobilized from the resorbed bone matrix, supporting tumor cells’ survival and proliferation. In turn, the growing tumor releases pro-osteolytic factors, fostering further osteolysis [40]. Since osteoclast differentiation and maturation are the critical processes in the pathogenesis of osteolytic lesions, several studies have demonstrated the involvement of the OPG–RANK–RANKL axis [41].

It has been shown that tumor cells secrete factors that directly or indirectly stimulate osteoclast formation and survival. Among tumor-secreted pro-osteolytic molecules, there are PTHrP, Interleukin-6 (IL-6), IL-1, Tumor Necrosis Factor α (TNFα), IL-8, IL-11, M-CSF, TGF-β, Vascular Endothelial Growth Factor (VEGF), Matrix metalloproteinases (MMPs), and prostaglandins [40].

PTHrP resembles PTH, sharing eight out of the 13 initial amino acids at the N-terminus, and binds to the PTH receptor type 1, known as PPR, expressed by osteoblasts, osteocytes, renal tubular cells, and tumor cells [42]. In bone metastasis, PTHrP, released by the tumor cells, acts in an autocrine manner, contributing to tumor proliferation and apoptosis resistance, and in a paracrine way, stimulating osteocytes and osteoblasts to secrete RANKL [43].

Prostate, breast, and colon cancer cells release high levels of IL-6 and also express the IL-6R/gp80 and gp130 receptor subunits. These features allow the creation of autocrine stimulation, enhancing the proliferation and survival of the tumor cells [44]. IL-6 has many effects on bone tissue: (1) It induces the expression of RANK-L from stromal cells and osteoblasts [45]; (2) It stimulates tumor cells to express PTHrP, IL-8, IL-11, RANK-L, and Cyclooxygenase 2 (Cox-2), inducing bone resorption [44,46]; (3) It inhibits osteogenesis regulated by Wnt [47]; (4) It stimulates the activity of estradiol 17 β-hydroxysteroid dehydrogenase, countering the protective role of the estrogens on bone and enhancing bone resorption and hypercalcemia in breast cancer patients; (5) It reduces the expression of genes like type II collagen and aggrecan, reducing bone formation. All together, these effects lead to tumor progression and reduction of the bone mass [44,48].

The levels of Interleukin 1b (IL-1b) are high in many cancers and are associated with a worse prognosis. Moreover, it was shown that the overexpression of IL-1b in non-metastatic prostate cancer cells promoted bone metastasis, whereas the knockdown of IL-1b impaired the bone progression of metastatic cells [49]. Moreover, IL-1 induces bone resorption by acting on osteoclasts directly after binding to its receptor, or indirectly through the stimulation of RANK-L and prostaglandin synthesis in the bone [50].

IL-8 is a proinflammatory CXC chemokine, secreted by monocytes, endothelial cells, osteoblasts, and tumor cells, and it can activate osteoclasts [51]. Cancer cells have been shown to directly produce IL-11 and to stimulate osteoblasts to secrete IL-11 [52], which in turn is known to suppress the activity of osteoblasts [53]. Kudo et al. showed that IL-11 is able to stimulate RANK-L-independent osteoclastogenesis, while other studies suggested that its osteolytic effect is due to its ability to stimulate RANK-L secretion from osteoblasts [54].

TGF-β is released from the bone matrix during bone resorption and it is secreted by tumor cells [55,56]. Yin et al. demonstrated that blocking TGF-β signaling by expressing a dominant-negative type II receptor serine kinase TGFβRII (DNTβRII) in MDA-MB-231 breast cancer cells inhibited the production of PTHrP by cancer cells and the osteolytic lesions in an animal model [57]. TGF-β stimulates the proliferation of tumor cells and has a dual effect on osteoblast cells: it acts on osteoblast precursors stimulating their differentiation and, if it binds to its receptor expressed by mature osteoblasts, it inhibits their function [58].

It was shown that metastatic breast cancer cells in bone express relatively high levels of VEGF and of VEGF receptors [59]. VEGF directly enhances osteoclastic bone resorption and survival of mature osteoclasts through binding to VEGFR1 and VEGFR2 [60].

MMPs are zinc-dependent proteases that play a major role in the regulation of the extracellular matrix through the degradation of its structural components. Even if tumor-associated MMPs do not directly digest mineralized matrix, they may participate in osteolysis [61]. Indeed, tumor MMPs can cleavage the collagen that covers the bone surface and stimulate bone resorption mediated by osteolytic factors. Another role of MMPs in osteolysis include cleaving membrane-bound Endothelial Growth Factor (EGF)-like growth factors that bind EGFR in osteoblasts in order to suppress OPG and increase the ratio of RANK-L to OPG to favor osteoclastogenesis [62].

Prostaglandin E2 (PGE2) is associated with inflammation, cell growth, tumor development, and metastasis [63]. Its binding to the receptor EP4 is important in osteolysis, since it induces the differentiation of monocytes into mature osteoclasts [64]. Moreover, Ohshiba et al. demonstrated that the direct contact between cancer cells and osteoblasts induces Cox-2 expression in the osteoblasts due to the activation of the NFκB–mitogen-activated protein kinase (MAPK) pathway. This activation stimulates the production of PGE2, that can also act in an autocrine manner, increasing the production of RANK-L by the osteoblasts [65].

### 3.2. Osteosclerotic Lesions

In osteoblastic metastasis, new woven bone deposition by osteoblasts is observed. The mechanisms leading to sclerotic lesions are based on increased osteoblast differentiation and function. Tumor cells secrete several factors that stimulate various steps involved in the differentiation, proliferation, and maturation of osteoblasts or in the inhibition of osteoclasts. Among these, there are Wnt proteins, Endothelin 1 (ET-1), BMPs, IGFs, IL-6, OPG, TGF-β, Urokinase, PDGF-BB, FGFs, Prostate-specific antigen (PSA), and VEGF [29,40].

Endothelin-1 is a potent vasoconstrictor that belongs to a family of three 21-amino-acid peptides [66]. Its expression was detected in osteoblasts, osteocytes, and vascular endothelial cells. The endothelins mediate their effects through endothelin A (ETA) and endothelin B (ETB) receptors, both expressed by osteoblasts. Endothelin-1 stimulates the proliferation of osteogenic cells; its effects on osteoclasts are still controversial [67]. ET-1 produced by tumor cells acts in an autocrine manner, enhancing the growth and invasiveness of a tumor, and in a paracrine way on bone cells [68].

Hiraga et al. suggested that IGF, released from the bone matrix or by tumor cells, increases proliferation and decreases apoptosis in breast and prostate cancer cells that are colonizing the bone [69]. These effects are mediated by the binding to its receptor IGF1R and the activation of the Akt and NF-κB pathways and lead to the development and progression of bone metastases. Moreover, IGF-I and -II stimulate bone formation, increasing the apposition of bone matrix by osteoblasts and, at the same time, decreasing collagen degradation [70].

PDGF-BB is a dimeric polypeptide growth factor. It affects several biological events associated with tumorigenicity, invasion, and distant metastasis, such as the promotion of cell proliferation, cell migration, and angiogenesis. It is an important osteotropic factor and is able to induce the migration and proliferation of osteoblasts [71].

Prostate cancer cells produce urokinase-type plasminogen activator (uPA) and PSA [30] that are able to affect osteoblast function. uPA binds to its receptor uPAR, expressed by osteoblasts, and induces cell proliferation and activation. uPA is able to cleave and activate TGF-β produced by the osteoblasts and, in turn, to regulate osteoblast function. Moreover, uPA hydrolyzes IGF-binding proteins (IGFBPs), thereby increasing free IGF levels [72]. Regarding the kallikrein serine protease PSA produced by prostate cancer cells, it is able to cleave PTHrP, thus reducing bone resorption, and IGFBP3, thereby freeing up IGF-I to bind its receptor and stimulate osteoblast proliferation [72].

The expression of FGFs is elevated in prostate cancer, and these factors can potentially act in either a paracrine or an autocrine manner [73]. FGFs regulate the osteoblastic response to bone metastasis. Indeed, FGF1 and FGF2 stimulate bone formation in vivo [74,75]. FGF2 induces the proliferation and differentiation of osteoblasts by upregulation of both RUNX2 and BMP and by modulation of the Wnt pathway [76].

In turn, activated osteoblasts form new bone and release numerous growth factors used by tumor cells to potentiate their survival and growth, establishing an amplifying loop between osteoblast bone formation and tumor progression.

In prostate cancer, bone metastases are primarily osteoblastic and form lesions adjacent to the tumor. Alkaline phosphatase and osteocalcin levels are elevated in patients with sclerotic metastasis [77]. However, many patients with prostate cancer also show osteolytic components in the bone lesions [78].

## 4. Antiresorptive Drugs

The use of antiresorptive drugs, including bisphosphonates (BP), represents the most effective treatment for patients with bone metastases to reduce SRE [79]. In 1968, Herbert Fleisch discovered the medical potential of bisphosphonates, which are currently used to treat patients affected by osteoporosis, Paget disease, osteogenesis imperfecta, and cancer-induced bone disease. BPs are chemically stable derivatives of inorganic pyrophosphate [80], a naturally occurring compound in which 2 phosphate groups are linked by esterification [81]. 

Two classes of bisphosphonates are known that differ for the structure and mechanism of action: nitrogen-containing BPs and non-nitrogen containing BPs. The first class includes pamidronate, alendronate, ibadronate, risendronate, and zoledronic acid, while non-nitrogen containing BPs include etidronate, clodronate, and tiludronate. Nitrogen-containing BPs are farnesyl diphosphate synthase inhibitors that act by impairing the mavelonate pathway, preventing the prenylation of GTPase signaling proteins important for cell vitality. Non-nitrogen-containing BPs kill osteoclasts, leading to the formation of cytotoxic metabolites [82].

BPs have a great affinity for bone mineral because they bind to hydroxyapatite crystals. Accordingly, the ability of these compounds to be entrapped into the skeleton is correlated to the availability of hydroxyapatite binding sites. They are preferentially incorporated where the bone turnover is high [81]. Several studies suggest that oral bisphosphonate treatment for postmenopausal osteoporosis reduces the incidence of invasive breast cancer. Moreover, the addition of zoledronic acid to conventional chemotherapy can reduce tumor size and ameliorate tumor responsiveness to chemotherapy. Indeed, pamidronate and zoledronic acid have been approved by the EMA and FDA for the prevention of SRE in adult patients with bone metastases from solid tumors and multiple myeloma [83].

However, a direct effect of bisphosponate on tumor cells still remains controversial. Jagdev et al. demonstrated that 10–100 µM zoledronic acid (ZA) for 72 h was required to induce apoptosis of breast cancer MCF-7 cells [84]. Koto et al. showed in vitro that ZA strongly inhibited the proliferation and induced the apoptosis of human fibrosarcoma cells [85]. Other papers suggested that the concentration of BPs at the tumor does not reach significant levels to kill tumors directly and that the observed effect on tumors in vivo is due to indirect effects [86]. It was demonstrated that zoledronic acid may also exert indirect anti-tumor effects by modulating the immune system. ZA is able to be internalized into CD45+ and F4/80+ tumor-associated macrophages (TAM) and reverse their polarity from M2 to M1. This is a very important finding, as M1 macrophages possess tumoricidal activity, supporting that TAM are a potential immune target of zoledronic acid therapy [87].

Another approach used to treat SRE in bone metastasis is RANK-L inhibition. It was already demonstrated that RANK-L is secreted not only from osteoblasts and osteocytes, but also from T and B cells [88]. The inhibition of RANK-L in bone metastasis could be important to reduce the burden of a tumor and reduce hypercalcemia [89]. However, of great interest are the effects of RANK-L inhibition directly on bone cells. Denosumab is a fully humanized IgG2 RANK-L antibody that is currently used for the treatment of osteoporotic patients. A single clinical trial suggested a possible role of Denosumab to counteract tumor growth [90]. Further studies suggest that Denosumab is more active than bisphosphonates to reduce bone tumor burden [89].

Besides these anti-osteoclast drugs, some agents are emerging that stimulate osteoblast activity, such as the antisclerostin monoclonal Romozumab [91].

## 5. Conclusions and New Perspectives

To understand the molecular and cellular mechanisms of bone metastasis onset and progression, further studies are required. Indeed, the identification of all molecules involved in this process could allow the discovery of new therapeutic targets for bone lesions. Recently, many studies are pointing the attention on the extracellular vesicles (EV)-mediated crosstalk between bone and tumor cells. Extracellular vesicles represent a heterogeneous population characterized by exosomes and microvesicles [92]. Although initially met with skepticism, their presence is now well established, and they represent an important way for cell-to-cell communication [93]. The content of EV is characterized by proteins, nucleic acids, and organelles; they can stimulate a target cell by ligand–receptor interactions, by endocytotic process, or by fusion, releasing their content directly inside the cytoplasm [94,95,96].

Several studies suggest that EV represent an important tool for tumor–bone communication. Indeed, it was demonstrated that tumor cells can use EV to prepare the bone for the growth of cancer cells and to stimulate osteoclast and osteoblast activity. In turn, bone cells can stimulate tumor cells proliferation by EV [29].

These studies suggest that the prompt identification of circulating extracellular vesicles released by primary tumor cells could be relevant for cancer diagnosis and therapy. However, the cellular and molecular mechanisms that regulate the release of vesicles from bone and tumor cells and their uptake by the recipient cells remain largely unknown. Hoshino et al. demonstrated that the proteomic profile and the integrin profile of tumor-derived EV are essential to predict the choice of organ to colonize [97]. Further efforts should be made to improve the methodology of EV isolation, and many studies are in progress to characterize EV content and to test their use as disease biomarkers. Indeed, despite the enormous therapeutic potential, the field of extracellular vesicles still requires further pre-clinical and clinical studies to clinically translate exosomes significance from the bench to the bedside.
